# Organizational culture and the individuals' discretionary behaviors at work: a cross-cultural analysis

**DOI:** 10.3389/fsoc.2023.1190488

**Published:** 2023-06-12

**Authors:** Pedro Fernandes, Rúben Pereira, Guilherme Wiedenhöft

**Affiliations:** ^1^Instituto Universitário de Lisboa (ISCTE-IUL), Lisbon, Portugal; ^2^Instituto de Telecomunicações (IT) - Instituto Universitário de Lisboa (ISCTE-IUL), Lisbon, Portugal; ^3^Institute of Economics, Administration, and Accounting Sciences at the University of Rio Grande, Rio Grande, Brazil

**Keywords:** organizational culture, organizational citizenship behavior, OCAI, cross-cultural, Kruskal–Wallis *H*-test

## Abstract

Stating “*how things are done here,”* organizations are defining their culture. Organizational Culture (OC) is the set of values, norms, goals, and expectations shared by all members of an organization that aids in improving their commitment and performance. On the organizational level, it impacts behavior, productivity, and long-term survival by influencing organizational capability. Due to employee behavior being a competitive differential, this study examines how specific OCs influence individual behavior. In particular, how the different cultures in the Organizational Culture Assessment Instrument (OCAI) affect employees' main dimensions of Organizational Citizenship Behavior (OCB). A descriptive-confirmative ex post facto research was conducted by surveying 513 employees from over 150 organizations worldwide. The Kruskal–Wallis *H*-test was used to validate our model. The general hypothesis was confirmed, showing that the predominant organizational culture type affects the level and the kind of OCBs individuals demonstrate. It is possible to provide organizations with a breakdown of their employees' OCBs based on their OC type and which changes they can make to their organization's culture to increase the employees' OCB and, consequently, the efficiency of their organization.

## 1. Introduction

Organizational Culture (OC) is a set of common values, norms, goals, and expectations shared by the organization's members (Quinn and Rohrbaugh, 1983). It is like an individual's identity, thus making them distinct from others (Appelbaum et al., 2004). A solid and well-defined OC is critical to increasing the company's productivity and long-term survival by enhancing the commitment and performance of its members (Hofstede, 1980; Sharoni et al., 2012; Jeong et al., 2019). It can be understood as organizational DNA, which determines individual and organizational behavior (Lockhart et al., 2020).

An organization's culture influences employee behavior in obvious and imperceptible ways (Erkutlu, 2011; Park C. H. et al., 2013). It impacts its members to develop attitudes and behaviors, such as attached or detached feelings and prosocial or antisocial behavior (Jain, 2015). Therefore, organizations pay more attention to behaviors within their OC since employees are more likely to meet their needs because of their high congruence with the OC (Tsai et al., 2012).

Although there is a broader range of similar concepts in the discretionary organizational behavior field, the Organizational Citizenship Behavior (OCB) was used in this study to understand the individual's behavior at work because of the amplitude given by its taxonomies, outcomes, and connection to the workplace environment (Organ, 2018). OCB refers to an individual's behavior that benefits the organization without being dependent on the organization's gratification system (Organ, 1988, 1997, 2015). They are spontaneous gestures of collaboration and protective actions to safeguard the organization and everything related to it (Rego, 2002). OCB is connected with job performance because citizenship behaviors are part of the spontaneous and innovative actions essential for effective organizations (Organ, 2018). The OCB comprises many dimensions that express and measure distinct behaviors and attitudes at work (Podsakoff and MacKenzie, 1997).

In addition to conveying what is needed, appropriate, and expected, OCs help generate an environment that encourages OCB development (Lockhart et al., 2020; Marcos et al., 2020). In recognition, many organizations are changing their organizational cultures to promote employees' creativity, self-discipline, and loyalty (Agarwal, 2016; Fernandes et al., 2021). However, as Limpanitgul et al. (2013) argue, although there have been numerous studies conducted on the development of OCB over the past few decades, only a small number of these studies have explicitly addressed the role of culture in understanding how OCB develops (Fernandes et al., 2021). Despite this, there is a consensus that the OCs can impact the degree to which employees perform citizenship behaviors (Jo and Joo, 2011).

By establishing their culture, organizations develop their way of doing things, and that influences employees' behavior and, consequently, OCB manifestations (Biswas and Varma, 2012; Ehrhart et al., 2015; Desselle and Semsick, 2016; Setyaningrum, 2017; Susita et al., 2020). According to Song et al. (2009), different OC types can result in other relationships employees perceive, yielding different responses. In cultures where people identify more closely with their profession and organization, they feel more satisfied and perform more OCBs directed to the organization (Conscientiousness, sportsmanship, civic virtue) (Lopez-Martin and Topa, 2019; Tagliabue et al., 2020). In contrast, in team-oriented cultures where employees feel they have a trust-based bond with their colleagues, they are more likely to perform individual OCBs (Altruism, Courtesy) (Lopez-Martin and Topa, 2019; Yin Yin Lau et al., 2020).

Following the literature that validates the relationship between OC and OCB in different ways, this study focuses on the premise that different cultures will affect how employees show different OCB dimensions, being able to predict which behaviors will be manifested depending on the existing cultural values. To validate these arguments and provide a deeper understanding of “*which”* behaviors and “*how”* different OC types impact them, this study works with the Organizational Culture Assessment Instrument (OCAI) model based on the findings of Fernandes et al. (2022). The OCAI model identifies and measures a company's prevailing and desired type of OC, which can affect how employees show distinct behaviors (Cameron and Quinn, 2011). Thus, this argument leads us to this study's problem: *The predominant organizational culture type affects the level, and the kind of OCBs individuals demonstrate*.

As a strategy to ensure sustainable business growth, organizations should try to adjust their own cultures to enhance the employees' OCBs, in turn affecting the performance of both the employees and the organization as a whole (Appelbaum et al., 2004; Agarwal, 2016; Huang et al., 2020). Organizations can utilize the OCAI model to identify the existing and desired types of OC from employees' perspectives (Cameron and Quinn, 2011). As a result of this study, organizations can learn how different OC types affect how employees demonstrate OCB and what changes will occur when moving from one OC type to another.

During this study, descriptive-confirmatory ex post facto research will be conducted, materialized in a survey to be answered by workers from various organizations worldwide. Validated instruments from previous studies will be used, followed by Kruskal–Wallis *H*-tests, to test our research question and hypotheses.

This article has the following structure: an introductory section introducing the research theme and its purpose. The theoretical background reviews and synthesizes OC and OCB concepts. Next, the theoretical model, constructs, and hypotheses are described, followed by a description of the applied method. The results section evaluates and explains the survey results using the Kruskal–Wallis *H*-test. The concluding section summarizes the findings, discusses the hypothesis tests' results and limitations, and suggests future research.

## 2. Theoretical background

### 2.1. Organizational citizenship behavior

Katz (1964) was the first to argue in favor of the discretionary behaviors' importance, writing, “*An organization which depends solely upon its blueprints of prescribed behavior is a very fragile social system.”* Since then, researchers have used a variety of different related concepts to measure and describe individual behavior, like OCB (Bateman and Organ, 1983; Smith et al., 1983), prosocial organizational behavior (Brief and Motowidlo, 1986), extra-role behavior (Van Dyne et al., 1995; Van Dyne and Lepine, 1998), organizational spontaneity (George and Brief, 1992; George and Jones, 1997), and contextual performance (Motowidlo et al., 1997). Podsakoff et al. (2000) analyzed these concepts, arguing that it is possible to find some differences between them besides being conceptually similar. Despite various concepts, OCB has been selected and employed in this research due to its widespread use in describing and measuring the influence of people's discretionary behavior in the workplace, which is supported by its dimensions and results (Organ, 2018).

OCB is characterized by the individuals' behavior who voluntarily benefits the organization and are not supported by the organization's gratification system (Organ, 2018). Although spontaneous and non-directly rewarded by definition, some schoolers have argued in favor of adding them to the formal evaluation system (Becton et al., 2008). Not being the rule or their purpose, literature shows us that individuals who show better OCB indicators are more frequently recommended for organizational rewards and promotions (Allen, 2006). In sum, OCBs are spontaneous gestures of collaboration, protective actions, and innovative behaviors that benefit organizations, are linked to job performance, and are essential for efficient organizations and sustainable business growth (Rego, 2002; Ehrhart et al., 2006; Organ, 2015). Due to the individual and organizational benefits, OCB's importance has grown and been a burgeoning concern for practitioners and schoolers (Podsakoff et al., 2014).

#### 2.1.1. OCB dimensions

Although we see in OCB's literature a consensus among scholars about citizenship gestures as prosocial acts of employees that benefit the organization (Smith et al., 1983), OCB's conceptions, dimensions, and constructs usually vary through the study's application and domain (Organ, 2015). OCB's research began mainly involving private sector businesses and addressing human resource management in Western countries' traditional fields (Smith et al., 1983). Over the years, thousands of studies in the literature presented us with more dimensions, areas of application, and studied regions (Podsakoff et al., 2014). Such dimensions are essential for measuring distinct OCB manifestations (Graham, 1991).

To our knowledge, Podsakoff et al. (2000) were the first to synthesize all the existent OCB dimensions in one study. At that time, they found 30 different OCB dimensions. The conceptualization of new dimensions continued to arise, and a few years later, this number is more than doubled (96), and the trend is that it will continue to increase (Fernandes et al., 2021). This study uses the five oldest and most used OCB dimensions (altruism, civic virtue, conscientiousness, sportsmanship, and courtesy). Because this research does not concentrate on specific geographical settings but rather on the OC factor, the selected dimensions are the ones that play better together and are often utilized throughout the organizational field and worldwide locations (Fernandes et al., 2021). Table 1 summarizes the definition of these dimensions.

**Table 1:** OCB dimensions.

**Dimension**	**Definition**
Altruism	Altruism is a helping behavior comprising all the voluntary actions that help others with a work problem
Conscientiousness	Conscientiousness is related to an excellent posture of going well-beyond minimum attendance levels, punctuality, housekeeping, conserving resources, and internal maintenance issues
Sportsmanship	Sportsmanship is the good behavior of an individual that focuses on what is right rather than wrong in an organization, tolerating the inevitable inconveniences and demands of work without complaint
Courtesy	Courtesy encompasses behaviors like being sensible of how one's behavior affects others to prevent work-related problems from happening
Civic virtue	Civic virtue represents individual involvement or concern in the organization's processes and life

### 2.2. Organizational culture

The OC incorporates elements that define the organization's functioning (Hofstede, 1980; Yergler, 2012). These elements are values, norms, objectives, and expectations commonly shared by the organization's members, differentiating them from each other (Quinn and Rohrbaugh, 1983). Personifying the concept, OC is the organization's personality and behavior over time (Rowlands et al., 2014).

Organizations have paid considerable attention to OC over the years because OC is regarded as a means of securing long-term survival and improving productivity (Cameron and Quinn, 2011). A solid and well-defined OC plays a vital role in the organization, improving its members' commitment and enhancing performance (Sharoni et al., 2012). It gives employees a comfortable feeling toward the organization, encouraging them to work collectively to achieve its goals (Rowlands et al., 2014). According to Aasi et al. (2014), organizations with better effectiveness indicators have a strong and positive culture.

OC can have many types of profiles, diverging in their combination of values (Cameron and Quinn, 2011). Despite having a prevailing one, no organization perfectly matches a single culture type, emphasizing various values (Quinn and Rohrbaugh, 1983; Wijesinghe et al., 2019). Song et al. (2009) argue that different OC types may enhance employees' perception of the relationship exchange and their response. Although slow, the OC constantly changes due to various internal and external influences on the organization (Rowlands et al., 2014).

#### 2.2.1. Organizational Culture Assessment Instrument (OCAI)

In previous research, it was possible to identify 17 distinct OC models by combining the concepts of OCB and OC (Fernandes et al., 2022). These models or profiles are responsible for identifying and measuring a company's prevailing type of OC, which can have distinct effects (Cameron and Quinn, 2011). Like other authors, this study used the Organizational Culture Assessment Instrument (OCAI) since it is one of the most widely utilized by academics linking these topics and practitioners with over 10,000 organizations using it (OCAI, 2022). OCAI was developed in 2011 by Cameron and Quinn (2011). Their studies were based on the Competing Values Framework (CVF), one of the most used OC frameworks (Quinn and Rohrbaugh, 1983).

For Cameron and Quinn (2011), OC is divided into four types, and organizations, besides having a dominant culture type, often develop their culture profile by mixing the four organizational culture types. OCAI enables the organization to examine its type of OC and the desired type by its employees. The desired culture type is achieved by the employee's perspective of how the organization should be in 5 years to be successful. Table 2 resumes these four types of OC and their main characteristics.

**Table 2:** OCAI model.

**Type**	**Definition**
Clan	It is commonly assumed that clan cultures are characterized by teamwork and employee development, that customers are best viewed as partners, that the organization fosters a humane work environment, and that management's primary objective is to empower and facilitate employees
Adhocracy	An adhocracy culture is characterized by a dynamic, entrepreneurial, and creative workplace where people take risks and stick their necks out. Leadership is visionary, innovative, and risk-oriented, and experimentation and innovation are the glue that binds an organization together
Market	Market cultures are results-oriented workplaces where leaders are hard-working producers and competitors, the glue holding the organization together is winning, and long-term concerns focus on achieving stretch goals
Hierarchy	The hierarchy culture defines a formal, structured work environment where procedures govern what people do. Effective leaders are skilled coordinators and organizers, where maintaining a smooth-running organization is essential, and the organization's long-term concerns are stability, predictability, and efficiency. As a result, formal policies and rules bind the organization together

## 3. Hypothesis development

### 3.1. Theoretical model

Based on the Systematic Literature Review results by Fernandes et al. (2022), it was possible to verify that researchers have interpreted how OC impacts OCBs differently over the years. There are references to OC as an antecedent of OCB (Biswas and Varma, 2012; Park S. M. et al., 2013; Tagliabue et al., 2020), a potential moderator of OCB (Erkutlu, 2011; Sharoni et al., 2012; Marcos et al., 2020), a predictor of OCB (Teh et al., 2012), an effective tool for performing OCB (Alsheikh and Sobihah, 2019; Jeong et al., 2019; Susita et al., 2020), and with OCB as part and one of the many OC measures (Desselle and Semsick, 2016; Desselle et al., 2017, 2018; Setyaningrum, 2017; Yuliusdharma et al., 2019; Jafarpanah and Rezaei, 2020). Nevertheless, there is evidence that OCs can influence employees' OCBs (Jo and Joo, 2011). This study starts from the premise that different cultures in the OCAI model will affect how employees show different OCB, being able to predict which behaviors will be manifested depending on the existing cultural values.

As shown in Table 2, the different culture types in the OCAI model indicate that employees may behave differently based on the dominant culture type. On the one hand, the clan and adhocracy cultures integrate employee interests, emphasizing trust, long-term support, and employee investment. Consequently, employees are willing to transcend their interests to pursue common goals, strengthening their OCBs (Song et al., 2009; Jeong et al., 2019). On the other hand, the hierarchy and market cultures are less likely to improve employees' OCBs (Song et al., 2009; Sharoni et al., 2012).

**Figure 1:**
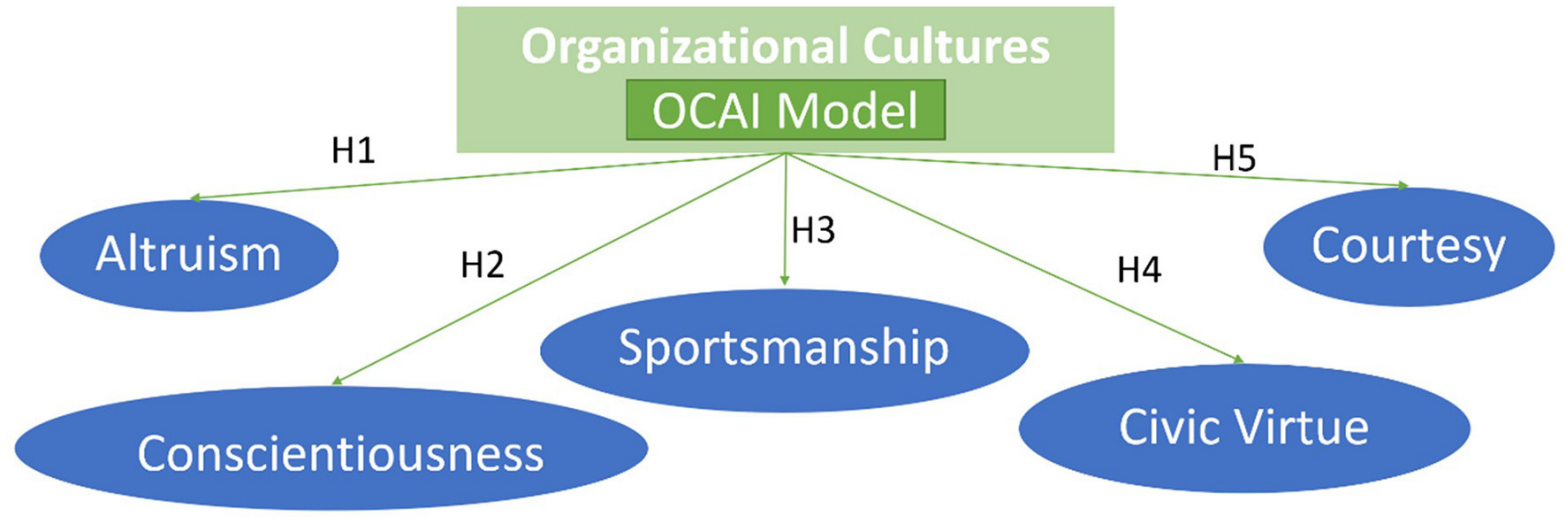
OC & OCB research model.

In a dominant hierarchical culture, employees are expected to follow standard operating procedures and rules rather than participate in decision-making. This expectation may not only lead to low levels of OCB but also affect the performance of organizations, which depend upon innovative and spontaneous activities outside their roles (Song et al., 2009). Similarly, in the dominant market culture, the orientation for results is generally related to low OCB levels associated with high turnover intention (Sharoni et al., 2012). Finally, these two cultures do not affect employees' OCB negatively, but adhocracy and clan culture is expected to lead to higher OCB levels (Youn et al., 2017; Jeong et al., 2019). In Figure 1, five hypotheses are proposed considering the OCAI Model's (Table 2) possible influence on the five OCB variables (Table 1).

Altruistic behaviors represent an employee's philosophy about helping others. Altruism is a stable characteristic of the individual representing those voluntary actions to help another person with a work problem (Goodman and Svyantek, 1999; Liu and Fellows, 2008). Thus, the perceptions of what happens in the organization may have less impact on this variable's performance than on a group and team-based culture (Goodman and Svyantek, 1999). The orientation toward innovation also favors these attitudes and behaviors (Lopez-Martin and Topa, 2019). Despite its presence in hierarchical organizations, it will be less expressive since few bureaucratic organizations rely on this behavior rather than rigid norms and formalized processes (Jeong et al., 2019). According to the OCAI model, two types of cultures motivate altruistic behavior. First, in a clan culture, the organization emphasizes the long-term benefit of human resource development by rewarding teamwork, participation, and consensus. Second, in an adhocracy culture, the organization encourages innovation, individual initiative, and freedom (Cameron and Quinn, 2011). Considering this, we hypothesize:

- **H1**: Individuals in an organization with a dominant adhocracy-oriented or clan-oriented culture demonstrate higher altruism levels.

Conscientiousness is a citizenship behavior toward the organization (Organ, 1988). It represents the employee actions above the organizational minimum required, such as going beyond attendance and punctuality, which fits well with collectivist cultures (Liu and Fellows, 2008). Thus, these discretionary behaviors are more impersonal and target the organization. Consequently, employees' perceptions of the organization's current state may be more predictive since the organization's conduct may influence this behavior's performance (Goodman and Svyantek, 1999). Organizations with a support-oriented culture, well-defined norms, and procedures that adapt continuously to the external environment are likelier to exhibit conscientiousness in their employees (Park S. M. et al., 2013; Jeong et al., 2019; Lopez-Martin and Topa, 2019). The OCAI model, in the form of market or hierarchical cultures, shows how organizations enhance formal rules and policies to remain united, caring about the security and stability of their employees and highlighting the external relations that drive competitiveness and the achievement of results (Cameron and Quinn, 2011). Essential points in the positive influence of conscientiousness allow us to write the following hypothesis:

- **H2**: Individuals in an organization with a dominant market-oriented or a hierarchy-oriented culture demonstrate higher conscientiousness levels.

Organ (1988) defined sportsmanship as the free will to tolerate work's inevitable impositions and inconveniences without complaining. As part of the behaviors that favor the organization, contrary to expectations, there are no references to a significant favorable influence of the hierarchical culture where the institutionalization of organizational norms and procedures is focused (Cameron and Quinn, 2011). However, looking more broadly at sportsmanship, we can find references to other organization members' behaviors. An employee with high sportsmanship will not complain when others bother him. For the workgroup to succeed, he will be willing to sacrifice his interests for the good of the workgroup. He maintains a positive attitude even when things do not go his way and do not offend others when his suggestions and ideas are not followed (Podsakoff et al., 2000; Liu and Fellows, 2008). Thus, sportsmanship will be positively affected by team-based cultures, like clan culture, where the task interdependence of the teams is high (Ehrhart et al., 2015; Yuliusdharma et al., 2019). Leading us to the following hypothesis:

- **H3**: Individuals in an organization with a dominant clan-oriented culture demonstrate higher sportsmanship levels.

Civic virtue represents an interest or commitment to the organization as a whole, and it is demonstrated by a willingness to participate in its governance and pursue its best interests even at high personal cost (Organ, 1988; Podsakoff et al., 2000; Liu and Fellows, 2008). This behavior and attitude are usually linked to a market culture, where the quest is to achieve high levels of organizational effectiveness and productivity in the long term (Park S. M. et al., 2013; Jeong et al., 2019).

Market culture is based on long-term focus, driving competitive actions to accomplish measurable goals and objectives, creating in individuals the will to participate, and helping the organization to develop commitment and emphasis on winning (Cameron and Quinn, 2011). Thus, the following hypothesis is drawn:

- **H4**: Individuals in an organization with a dominant market-oriented culture demonstrate higher civic virtues levels.

Courteous behaviors present a gesture toward others, helping them prevent work-related problems, for example, notifying them before acting in a way that may affect them (Liu and Fellows, 2008). Some clan and hierarchical cultures favor this behavior (Park S. M. et al., 2013). First, in a team-based culture, interdependence on the team creates a proper environment for providing extra help to co-workers. In a hierarchical culture, the emphasis on rules and regulations will enhance the demand and help to control and stabilize (Cameron and Quinn, 2011). That leads to this hypothesis:

- **H5**: Individuals in an organization with a dominant clan-oriented or hierarchy-oriented culture demonstrate higher courtesy levels.

### 3.2. Research method

**Figure 2:**
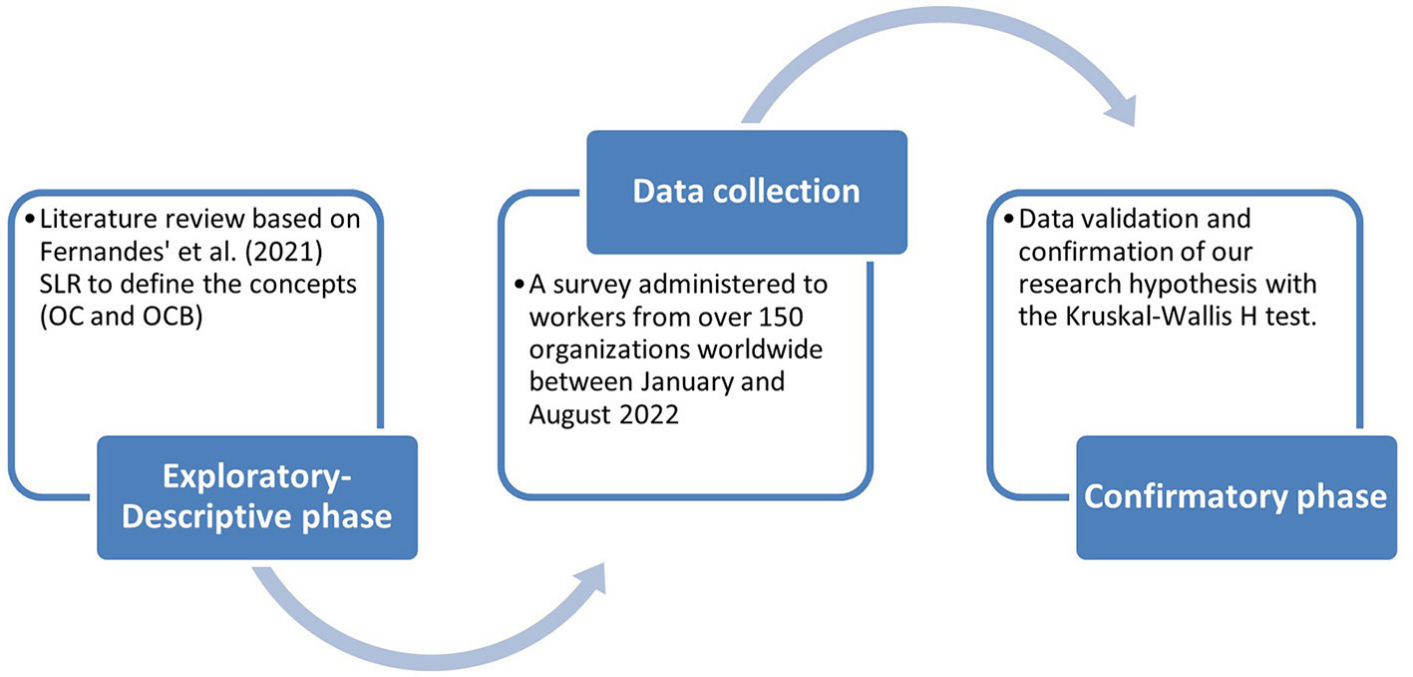
Research design.

This section describes the methodology for conducting the study, the instruments used, and the characteristics of the sample. This study takes a functionalist view of knowledge, seeking to understand society from a perspective that can guide organizations (Deetz, 1996). The research approach used in this study is a cross-sectional survey design based on descriptive-confirmatory ex post facto research. This approach involves collecting data from a sample of individuals simultaneously to describe the relationship between variables of interest (Venkatesh et al., 2013). Figure 2 resumes the research design.

Due to the nature of this study, survey research is an appropriate method to analyze a population sample and provide useful results (Glasow, 2005). A survey instrument is a measurement object that links a study's objectives with measurable variables to normalize and control the data for accuracy and reliability (Visser et al., 1986; Ponto, 2015). A non-probability convenience sampling technique was employed in this research to easily access conveniently available individuals willing to participate in the study that met the inclusion criteria (Taherdoost, 2016). Further generalization should consider the characteristics of this sample that aims to give useful information about the relationship between OC and OCB in a wide range of organizations worldwide. There were no specific rules for companies that want to participate, such as the type of industry or region, to ensure that a broader range of organizations and individuals is represented.

The questionnaire used comprises two instruments already tested and validated in the literature. The first one operationalizes the OCB model with 20 questions. The instrument consists of four questions for each of the five OCB dimensions (Podsakoff et al., 1990; Moorman, 1993; Lin et al., 2010). Secondly, the OCAI instrument was operationalized with four questions for each of the six dimensions of culture: Dominant characteristics, Organizational leadership, Management of employees, Organization glue, Strategic emphases, and Success criteria (Cameron and Quinn, 2011). This questionnaire will allow readers to engage in a multicultural study, enabling them to comprehend how an individual's OCB varies depending on the predominant OC.

A survey instrument was developed to gather information, and it was administered to workers from over 150 organizations worldwide between January and August 2022. The list of organizations invited was obtained through the authors' connections and network of contacts and from multiple pools online with sets of companies. After analyzing the company profile, the companies were individually invited via LinkedIn message, email contact, or contact form. The companies were chosen based on a broader aspect of the research in which this study is included and is connected with the existence of information technology governance policies. Despite that, no specific criteria for companies participating in this study, such as type of industry, were established. The only requirement was that the employees had to work at the company for at least 1 year to participate to ensure they understood the existing OC. Since the study focuses on the OC factor and its aimed to group results based on the perception of the predominant culture type by the individuals, the representation of regions in the survey results depends entirely on the company location and not on a question of representativity.

After the approval was given by the organizations participating in the study, an individual questionnaire was sent to each organization that was then responsible for distributing it internally to be responded to by its members. The questionnaires were sent online, and it was ensured on the first page of the questionnaire that the respondents had no obligation to respond and that no personal data would be saved, guaranteeing a voluntary and anonymous response. The questionnaires were created using LimeSurvey. This tool was selected because it allows the creation of unlimited surveys, the collection of an unrestricted number of results, and offers advanced options like question groups and different question types (Engard, 2009). A total of 557 surveys were completed at the end of the data collection process. Due to ethical considerations and the organizations' request, the questionnaires were anonymous, meaning participants could not be identified.

The data were analyzed in three phases. Firstly, the author had to ensure the research theme was familiar to the survey respondents. As a second condition, respondents should be employees of the organization for at least 2 years or have a minimum of 1 year of professional experience. From these two steps, seven answers were excluded. Finally, the data were examined to exclude missing values, suspicious response patterns, and outliers (Hair et al., 2017). Since the answers were all mandatory, finding any missing values was impossible. The standard deviation (SD) technique found 35 answers with suspicious response patterns such as straight-lining, diagonal-lining, and alternating extreme poles. These answers were removed from the data set. As a last examination, searching for outliers enabled us to delete two more answers from the dataset. The data cleansing procedure yielded 513 reliable results, corresponding to 92% of the survey results. Table 3 shows the respondents' profiles.

**Table 3:** Respondents' profile.

**Social-demographic variables**		**Frequency**	**Percentage (%)**
Gender	Male	280	55
	Female	233	45
Age	18–25 years	38	7
	26–35 years	144	28
	36–45 years	160	31
	46–45 years	128	25
	>55 years	43	8
Education	High school	39	8
	Bachelor's	180	35
	Master's	211	41
	PhD	48	9
	Other	35	7
Function	IT professional	88	17
	Human resources	10	2
	Director	57	11
	Manager	102	20
	C-level	38	7
	Other	116	23
	NA	102	20
Region	Africa	9	2
	Asia	15	3
	Europe	322	63
	Latin America and the Caribbean	63	12
	Middle East	16	3
	North America	45	9
	Oceania	43	8
Predominant OC type	Clan	201	39
	Hierarchy	130	25
	Market	99	19
	Adhocracy	66	13
	NA (all equal)	17	3

## 4. Results

This section evaluates the hypotheses formulated in the previous sections based on the survey results. As the Shapiro-Wilks test shows, it is impossible to determine the sample's normality (Sig. < 0.001) (Hair et al., 2018). Considering the lack of normality of the data, a Kruskal–Wallis *H*-test was used (Hamdollah and Baghaei, 2016). This test is a non-parametric version of the one-way Factorial analysis of variance (ANOVA) and is used in cases like ours (Abu-Bader, 2021). By applying the Kruskal–Wallis *H*-test to respondents' mean levels of behavior based on their predominant OC Types, we will see if there is a statistically significant difference between them.

The results were evaluated using the IBM SPSS 28 Statistics Software (Statistical Package for the Social Sciences). In the first step, we applied the Kaiser–Meyer–Olkin (KMO) and the Bartlett sphericity test to check the sample adequacy (it is ensured by a KMO statistic above 0.5) (Field, 2005). As highlighted in Table 4, the KMO of the analysis is 0.763 (“good” according to Field, 2005). Plus, Bartlett's test of sphericity, the approximate Chi-square is 1,519.318 with 15 degrees of freedom, which is highly significant at this level (Sig. < 0.001 according to Field, 2005). This information makes it credible to conclude that the data is suitable for factor analysis.

**Table 4:** Kaiser–Meyer–Olkin and Bartlett tests.

Kaiser–Meyer–Olkin measure of sampling adequacy		**0.763**
Bartlett's Test of sphericity	Approximate chi-square	1,519.318
	Degree of freedom	15
	Significance	0.000

**Figure 3:**
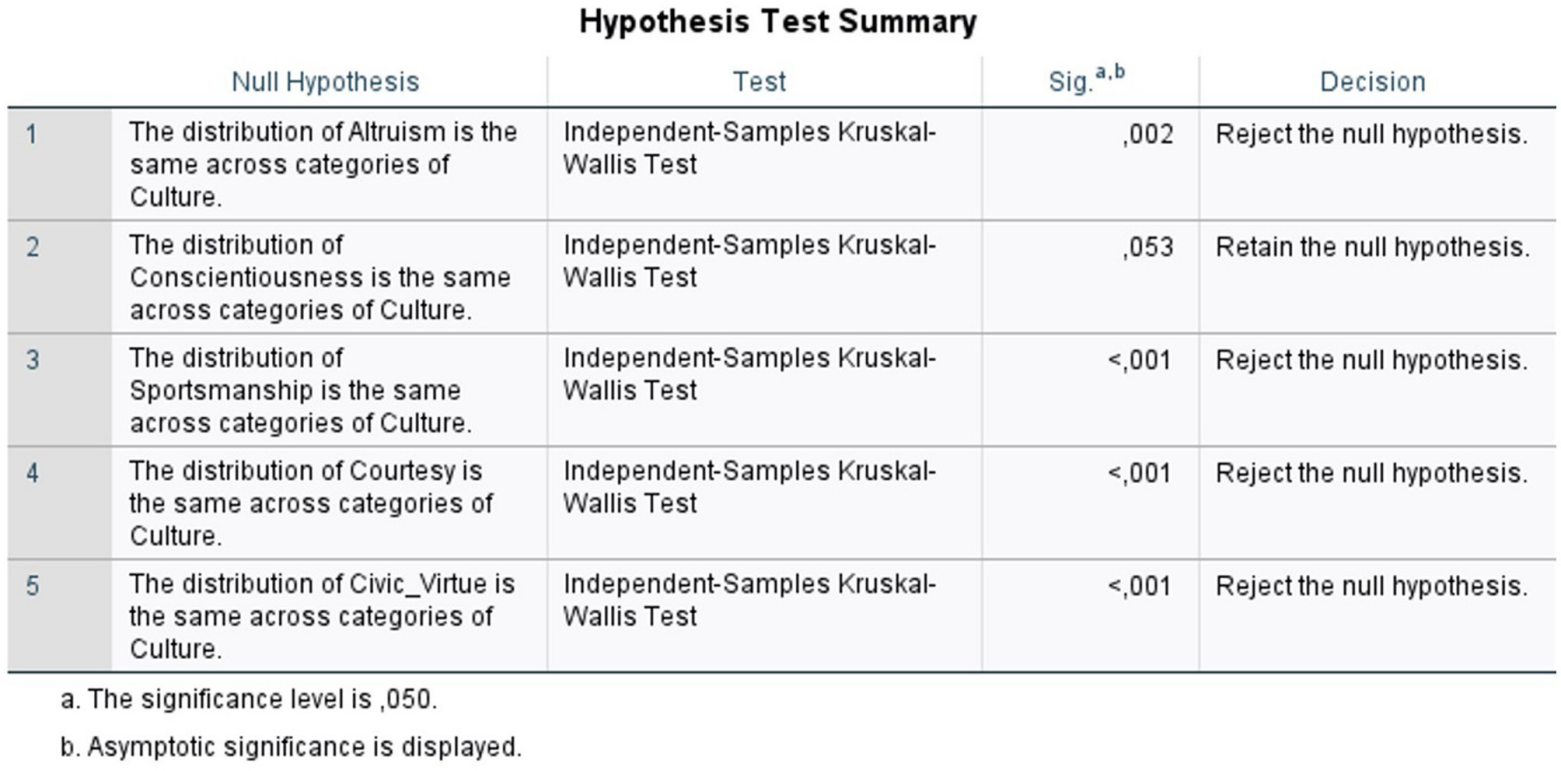
Kruskal–Wallis *H*-test hypothesis testing.

The first step in this analysis was determining each response's predominant culture type. Table 3 shows this data, and since 17 respondents could not provide a dominant culture type, they were not considered. Next, the data was entered into SPSS software, where the four indicators for each OCB dimension were merged by their means into one variable for each dimension. A Kruskal–Wallis *H*-test was conducted according to the Abu-Bader (2021) guidelines to determine if the employees' OCB were different for the four kinds of OC: Clan (*n* = 201), Adhocracy (*n* = 66), market (*n* = 99), and Hierarchy (*n* = 130). Based on the Kruskal–Wallis *H*-test, Figure 3 illustrates a statistically significant difference among the four cultural types for four OCB dimensions (*p* < 0.05). However, this variance was not statistically significant for conscientiousness behavior (*p* > 0.05) and was therefore ignored (Abu-Bader, 2021).

Following our analysis, Table 5 presents the *post hoc* Bonferroni correction for each pairwise comparison and the mean rank for each dimension. As a result of averaging these ranks for all observations within each sample, we can identify which cultures differ significantly and which have more influence on a particular behavior.

**Table 5:** Results of Kruskal–Wallis *H*-Test—OCB dimensions by culture type.

**Culture type**	***N***	**Mean rank**	**Clan**	**Adhocracy**	**Market**	**Hierarchy**	
**Altruism**							
Clan (H1)	201	272.06	–				H1—Partially confirmed
Adhocracy (H1)	66	266.36	5.729	–			
Market	99	217.26	54.827^*^	22.591	–		
Hierarchy	130	226.76	45.323^*^	21.487	−9.504	–	
**Conscientiousness**							
Clan	201	All indicators have a non-significant difference (*p* > 0.05)					H2—Not confirmed
Adhocracy	66						
Market (H2)	99						
Hierarchy (H2)	130						
**Sportsmanship**							
Clan (H3)	201	281.08	–				H3—Partially confirmed
Adhocracy	66	277.79	3.294	–			
Market	99	221.74	59.340^*^	56.045	–		
Hierarchy	130	203.63	77.451^*^	74.157^*^	18.112	–	
**Civic virtue**							
Clan	201	280.17	–				H4—Not confirmed
Adhocracy	66	284.58	−4.414	–			
Market (H4)	99	216.81	63.356^*^	67.770^*^	–		
Hierarchy	130	205.35	74.823^*^	79.237^*^	11.467	–	
**Courtesy**							
Clan (H5)	201	272.64	–				H5—Partially confirmed
Adhocracy	66	286.91	−14.265	–			
Market	99	207.32	65.326^*^	79.591^*^	–		
Hierarchy (h5)	130	223.03	46.614^*^	63.878^*^	−15.713	–	

* p < 0.05.

As shown below, the altruism dimension shows a statistical significance difference between Market and Clan (test statistic = 54.827, *p* < 0.05) and Hierarchy and Clan (test statistic = 45.323, *p* < 0.05). In contrast, there was no significant difference between Clan and Adhocracy culture (test statistic = 5.729, *p* > 0.05) and the other pairwise comparisons. Regarding the level of altruism shown by the different cultures, it can be stated that the market culture reported a significantly lower level of altruism (mean rank = 217.26) than its different culture types (Hierarchy = 226.76; Adhocracy = 266.36; Clan = 272.06).

Sportsmanship, civic virtue, and courtesy behaviors should be analyzed as altruism by seeing if there are statistical differences between their pairs (^*^) and the level of behavior shown by their rank power. In contrast, the conscientiousness behavior results are irrelevant since all indicators have a non-significant difference (*p* > 0.05).

## 5. Discussion

This study aimed to examine how different OC types affect the behavior of individuals in organizations worldwide. In Figure 3, it is shown that there is a significant relationship between the different OC types and four dimensions of the OCB, as indicated by *p*-values under 0.05. As it represents the majority of the dimensions in the study, this confirms the study's general hypothesis that the predominant organizational culture type affects the level and the kind of OCBs individuals demonstrate. Below are the five hypotheses of which our general hypothesis consists:

- **H1:** This hypothesis aimed to demonstrate that members of organizations with a dominant adhocracy-oriented or clan-oriented culture display higher altruism. The results of our study partially supported this hypothesis. As one can see, the clan culture is confirmed as showing higher levels of altruism as it shows the highest mean rank (272.06) and a significant difference with the hierarchy and market (*p* < 0.05) cultures. In contrast, this result is not confirmed for the adhocracy culture, which has the second-highest mean rank (266.36), but that is not significantly relevant considering the other OC types (*p* > 0.05). The concept of altruistic behavior represents an employee's philosophy about helping others, and our findings allow us to propose that teamwork and humane work environments foster it significantly. In contrast, organizations that are committed to experimentation and innovation are not able to make a significant impact on altruistic behavior. This insignificant impact may be likely because team membership is temporary, and no clear map can be drawn to identify communication channels (Cameron and Quinn, 2011).
- **H2**: The primary purpose of this hypothesis was to demonstrate that conscientiousness levels are higher in an organization with a dominant market-oriented or hierarchy-oriented culture. This hypothesis was rejected since the rank means between the different culture types was not significantly different (*p* > 0.05). This result can support previous research that argued for abandoning some behaviors as conscientiousness because their findings indicated that, in the eyes of managers at least, conscientiousness was an essential part of the behavior expected at work (Paillé, 2009; Fernandes et al., 2021).
- **H3**: In this hypothesis, sportsmanship levels are expected to be higher in organizations with a dominant clan-oriented culture. According to our findings, this hypothesis is partially supported. As one can see, the clan culture is confirmed as showing higher levels of sportsmanship as it shows the highest mean rank (281.08) but with no significant difference with the adhocracy culture (*p* > 0.05) in contrast with the hierarchy and market (*p* < 0.05) cultures. It has been confirmed that clan cultures associated with a friendly and caring working environment positively affect sportsmanship, which helps to accept and tolerate work's inevitable annoyances and inconveniences. In addition, adhocracy's emphasis on individuality, risk-taking, and anticipating the future affects sportsmanship as positively as clan culture.
- **H4**: This hypothesis was aimed at showing that employees in organizations dominated by market culture are more likely to display civic virtue. Our findings reject this hypothesis since the highest civic virtue levels are delivered in the adhocracy (mean rank = 284.58) and clan (280.17) cultures. By participating in the organization's governance and pursuing its best interests, even at a high personal cost, civic virtue indicates an interest or commitment to the organization. These findings allow us to understand the unanticipated impact of centered power and authority concentrated in market cultures on people's willingness to sacrifice themselves for the organization. In contrast, it allows us to conclude that civic virtue levels are high in adhocracy and clan cultures, where power is transferred from individual to individual or team to team (Cameron and Quinn, 2011).
- **H5**: This hypothesis aimed to show that individuals working in organizations with clan-oriented or hierarchy-oriented cultures exhibit higher levels of courtesy. As a result of our study, it is almost impossible to accept this hypothesis. It is evident from the results that employees from adhocracy cultures are more courteous (mean rank = 286.91) but that their levels of courtesy are like those of clan employees (*p* > 0.05), which allows us to assume that clan employees also possess elevated levels of courtesy. For the hierarchy culture, which has the third-highest mean rank (223.03), it is difficult to confirm this result. Courteous behavior helps others prevent work-related problems, and these results demonstrate that it is more common in clan cultures where success is based on internal climate and concern for people and in adhocracy cultures where people take risks and stick their necks out. In contrast to what was expected, the hierarchy culture's clear lines of authority, standardized rules and procedures, and control do not increase courtesy.

## 6. Conclusions

**Figure 4:**
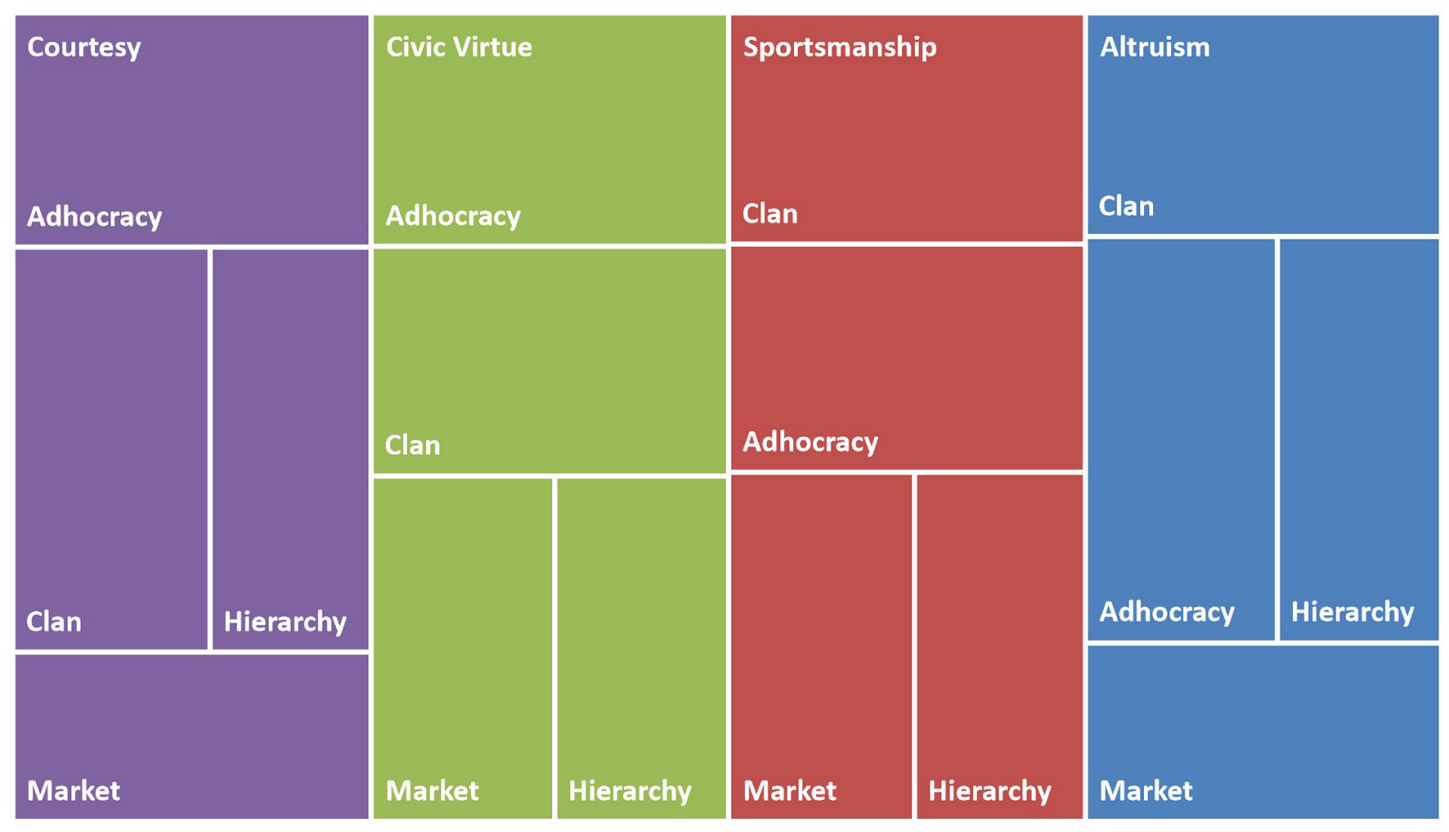
OCB dimension level by culture type.

These results allow us to draw some general conclusions. One can confirm the model's validity proposed in this study by looking at the theoretical path. Although some results were unexpected, detecting distinct levels according to the predominant culture type across all OCB dimensions except conscientiousness was possible. The results of this study lend credence to the theory that states that an organization's culture influences its members' attitudes and behaviors, such as attached or detached feelings and prosocial or antisocial behaviors (Jain, 2015). Additionally, Podsakoff et al. (1997) empirically demonstrated that organizations with higher OCB have superior effectiveness indicators, which suggests that organizations that move toward a clan or adhocracy culture will improve their employees' OCB and, consequently, their organizational effectiveness. Organizations that use the OCAI model to understand their cultural profile will know how likely their employees are to show different attitudes and behaviors and what will happen if they change their culture to another type. Figure 4 synthesizes the theoretical findings by giving an overview of the effect of the various culture types on the different behaviors with a treemap divided by OCB dimension and culture type. The OCB dimensions are separated by color, and the culture type where these behaviors are more prominent to be shown is ordered from the top to the bottom and the left to the right by the larger rectangle.

From a managerial and practical point of view, the results of this study will help organizations that want to foster behaviors and attitudes like helping others, acting responsibly, participating in organizational governance processes, and preventing work-related problems among their employees. Organizations must adapt and change to create a warm and supportive work environment (clan culture) or foster innovation and creativity by rewarding workers for taking risks and thinking outside the box (adhocracy culture) to promote these OCBs. Managers can support this change in two ways. On the one hand, they can create activities like team-building exercises and training programs that foster a culture of trust, open communication, and collaboration, rewarding and recognizing teamwork and cooperation. On the other hand, they can develop a change management mindset to create a dynamic environment where risk-taking, creative solutions, and innovation are encouraged, rewarded, and recognized. It is essential to develop cultural awareness at a management level to foster these behaviors and attitudes, enhancing organizational effectiveness.

As a result of this research, it is possible to show that organizations with clan or adhocracy cultures will show increased OCB indicators and better company outcomes. As outcomes of high levels of OCB among employees, they will benefit from enhanced career possibilities, stronger relationships at work, and greater job satisfaction. Organizations should foster this dynamic workplace culture that motivates employees to go above and beyond their job requirements, enhancing the overall mood and creating opportunities for them in the workplace, leading to better organizational outcomes as part of the process. Since the culture is constantly changing due to various internal and external influences on the organization, the significance of the results of this study should support the direction of this change and motivate organizations to provide a workplace that encourages workers to act in ways that are mutually beneficial to themselves and the company as a whole (e.g., by emphasizing values that characterize the clan and adhocracy cultures). Ultimately, this change may help organizations to reduce turnover intentions and increase their hiring capabilities since employees will choose to work for companies whose values are similar and have a positive work environment.

## 7. Limitations and future research

Even though the results of this study are relevant, they should be interpreted with caution due to some constraints. Firstly, the sample size is not particularly large for the adhocracy (*n* = 64) and market (*n* = 99) cultures, which makes generalizations difficult compared to the clan culture (*n* = 201). Although this limitation existed, the study could meet the minimum requirements for the research techniques. Secondly, not all regions are significantly represented in the survey results. This limitation was acknowledged but ignored since the study focused on comparing different OCB dimensions among cultures, ignoring the national culture factor. Therefore, further studies must better understand the impact of national cultures on the relationship between the different OC types and how employees manifest their OCBs. The influence of national cultures on how individuals perceive the existing OC, the effects on how different kinds of companies (regional, multinational) build and change their culture, and how individuals are predisposed to show different behaviors and attitudes depending on their national culture should be considered (Van Muijen and Koopman, 1994; Owusu Ansah and Louw, 2019; Fernandes et al., 2021).

More results may be collected in future studies to improve the conclusions of this study. It should be recognized that the organization's culture can affect individual behaviors differently depending on the individual's role in an organizational hierarchy. Recently in the literature, 96 OCB dimensions were found (Fernandes et al., 2021). This study used the five oldest and most used. The following authors should try to understand if these results are extensible to more dimensions. If the relationship between clan and adhocracy cultures and the distinct kinds of OCB dimensions remains significant, further research should investigate the main reasons for this outcome. One clue can be that markets or hierarchies have centralized power or authority relationships in contrast with the clan and adhocracy cultures. Also, understand which combinations of cultures make the clan and adhocracy cultures the best toward the different OCBs.

## Data availability statement

The raw data supporting the conclusions of this article will be made available by the authors, without undue reservation.

## Ethics statement

Ethical review and approval was not required for the study on human participants in accordance with the local legislation and institutional requirements. The patients/participants provided their written informed consent to participate in this study.

## Author contributions

All authors listed have made a substantial, direct, and intellectual contribution to the work and approved it for publication.
